# COVID-19 and the plight of migrants in India

**DOI:** 10.1136/postgradmedj-2020-138454

**Published:** 2020-08-12

**Authors:** Karthikeyan P Iyengar, Vijay Kumar Jain

**Affiliations:** Department of Orthopaedics, Southport and Ormskirk NHS Trust, Southport, UK; Orthopaedics, Atal Bihari Vajpayee Institute of Medical Sciences, Dr. Ram Manohar Lohia Hospital, New Delhi 110001, India

To contain the spread of the novel Coronavirus SARS-CoV-2 (COVID-19) outbreak, the Indian government initiated the largest national lockdown in the democratic world on March 2020. Initially announced to last until April 14, 2020, currently, it is in phase 4 which ended on May 31, 2020. The lockdown has severely affected the movement of people, disrupting daily life and access to healthcare facilities in India.^1 2^

As of June 9, 2020, India has a total of 266 598 confirmed cases and 7471 confirmed deaths attributed to COVID-19.^3^ In a population of more than 1.3 billion, the COVID-19 pandemic has had far-reaching consequences beyond the spread of the disease itself and efforts to quarantine it, including political, cultural, and social implications.

One of the consequences of lockdown measures in the country has led to an unprecedented migration of workers and families from large urban centres to rural India. For decades, millions of workers have migrated from their rural homes and villages to urban cities, looking for opportunities and livelihoods. Migrant labours in India from rural areas work as domestic help, in construction site, factories, industries, agriculture, etc, for better employment, better wages and better standard of living. The Indian government’s sudden enforcement of lockdown following a 14-h Janata curfew on March 22, 2020 immediately disadvantaged already vulnerable populations as it restricts people stepping out from their homes. All transport—roadways, airways and railways—were suspended, including hospitality industries, educational institutions, and industrial units. As the factories and workplaces closed down, millions of migrant workers had to deal with loss of income, food shortages and an uncertain future. The scale of this issue varies from state to state or city to city, but has caused widespread disruption. With no money, no job, unsure when the lockdown will finally end, the migrant workers had no other option than to return back to their villages. Their massive migration from working states has formed a humanitarian and health security challenge and an exceptional logistical nightmare.^4^

This instigated the next problem for them. How do they reach home? With road and rail transport links still suspended, walking back was the only option and they initially took to the road. Images of marching migrant workers most of them left with nothing but keen to reunite with their families back home. People have undertaken hazardous journeys, sometimes walking up to 1000 km with no money to spend and often without food for days together. Many were arrested by law enforcement officials for violating the lockdown, some died due to exhaustion or accidents on the roads.

The Social media (Facebook, Twitter, WhatsApp) are currently flooded with heart-breaking visuals that show migrant workers walking barefoot, foot with deep ulcers, women carrying their children on their waist, mother dragging baby on a suitcase, girl riding bicycle hundreds of kilometres carrying her father, etc. These visuals raise questions on the arrangements made by the state governments for the well-being of these migrant workers who have been stranded for many days in heatwave without food, water wages or shelter since the lockdown was started.

The state government started arranging buses to take migrants back to their villages for free. But these were heavily oversubscribed with migrants hanging on footboards and climbing on rooftops to find a place. Some group of migrants tried to hire people carriers, however returning migrants faced other danger—Indian roads! Road crash is 10 times more likely to kill someone than getting infected by the coronavirus in India. According to Aarogya Setu, the Government of India mobile contact tracing COVID-19 as off May 23, 2020 the current fatality rate of coronavirus infected people is about 3.2%.^5^ Data based on media reports by Save Life foundation, a non-profit non-government organisation focused on road safety across India, 381 people have died in 1200 road accidents.^6^ This translates to a fatality rate of nearly 32%. Thirty per cent of these victims were migrants travelling back home. The common cause of accident was speeding.

On May 1, 2020, the Indian government introduced special ‘Shramik Special’ trains from many districts in the country for migrants. Since then, Indian railways have ferried over 3 million migrant workers by more than 2050 Shramik special trains; however, a large number of migrants (70%) are still waiting for trains to return them home. Migrants have to register to book a place, undergo thermal screening prior to boarding for onward journeys. Unfortunately, an extraordinary rush and shortage of trains are resulting in endless waits for the migrants at the screening centres. At least 24 pregnant women have given birth during their journey between May 1 and May 21, 2020.

For many migrant workers who have crisscrossed the country desperately trying to return home, homecoming has been bittersweet. They stare at another crisis. With significantly reduced work opportunities (the very reason for them to migrate) and trying to come to terms with the labelling of ‘virus carrier’ stigma has caused a great deal of anxiety and associated violence has been reported. Migrants thought that going back to their hometown, they could return to farming or take up employment in the Mahatma Gandhi National Rural Employment Guarantee Act (MGNREGA) 2005 scheme.^7^ State governments are trying various job initiatives to support these returning workers, but it has been extremely difficult to formulate schemes and accommodate thousands of workers returning to their villages over such a short interval.

The overarching concern is workers returning back from cities and their places of work is the risk of spread of viral infection from the urban ‘hotspots’, to the rural villages. With thousands still returning home to their villages every day, the rural system does not have infrastructure to put all of them in institutional quarantine. In addition, there are shortages in facilities providing COVID-19 testing. The principal of ‘test, track and contact tracing’ to prevent the spread of coronavirus appears to be difficult to achieve in these circumstances. The apprehension is an explosion of COVID-19 cases in the villages and a real prospect of ‘second wave’ surge of the outbreak.

With chronic underfunding in the rural healthcare and economy, the pandemic has highlighted the failings of rural infrastructure.

The extraordinary migrant crisis due to COVID-19 is unparalleled since Indo-Pakistan partition of 1948. Similar to the fallout from the partition, this current crisis will leave a lasting legacy on future of India. It appears the COVID-19 pandemic has forced India to finally acknowledge the migrant!
